# Editorial: Psychosocial effects of isolation and fear of contagion of COVID-19 on the mental health of different population groups

**DOI:** 10.3389/fpsyg.2022.1011028

**Published:** 2022-09-12

**Authors:** María Cristina Richaud, Rubén N. Muzio, Viviana Lemos, Sebastián Urquijo, Gustavo Carlo

**Affiliations:** ^1^Consejo Nacional de Investigaciones Científicas y Técnicas, Buenos Aires, Argentina; ^2^Centro Interdisciplinario de Investigaciones en Ciencias de la Salud y del Comportamiento, Universidad Adventista del Plata, Libertador San Martín, Argentina; ^3^Instituto de Ciencias para la Familia, Universidad Austral, Buenos Aires, Argentina; ^4^Equipo GPS Salud (Grupo de evaluación y seguimiento del Personal de Salud), Buenos Aires, Argentina; ^5^Instituto de Biología y Medicina Experimental (IBYME)-Consejo Nacional de Investigaciones Científicas y Técnicas (CONICET), Buenos Aires, Argentina; ^6^Facultad de Psicología, Instituto de Investigaciones, Universidad de Buenos Aires, Buenos Aires, Argentina; ^7^Instituto de Psicología Básica, Aplicada y Tecnología, Universidad Nacional de Mar del Plata, Mar del Plata, Argentina; ^8^School of Education, University of California, Irvine, Irvine, CA, United States

**Keywords:** COVID-19, social isolation, fear of contagion, mental health, social groups, socio-political and economic context

The pandemic outbreak of COVID-19 has confronted us more than a health crisis, expanding the magnitude of its consequences as a human, economic, and social crisis and becoming a case of global disaster. Different conditions as the characteristics of the catastrophe and the socio-cultural context determined the impact of the catastrophe (Ozer et al., 2003; Porter and Haslam, 2005). In the current COVID-19 outbreak, several psychological problems, as well as consequences in mental health, as stress, depression, anxiety, and intolerance of uncertainty, have increasingly emerged throughout time while the disease continues to spread.

Necessary precautions to moderate the spread of the disease, such as isolation, produced an increase of people anxiety and stress even if they had not been infected with COVID. At the same time, being locked up for 2 weeks or more can be affected by the pressure on finances, the danger of unemployment, uncertainty about how to collect salaries and pensions, lack of social contact, apprehension toward the unknown, and worries for the health of oneself and others. In people with psychopathology these factors can have an even greater impact. Research on responses to previous pandemics such as SARS, Ebola, and H1N1 (Brooks et al., 2020) and recently COVID-19 (Dong and Bouey, 2020) indicated that it could cause long-term problems in the general population such as depression, high irritability, anxiety, family conflict, domestic violence, use of substances and alcohol, and Post Traumatic Stress Disorder.

Also it is important, as was mentioned earlier, to take the characteristics of each context into account when detecting the specific stressor that impact on psychological wellbeing. Indeed, different countries took particular health measures to face the pandemic based on consideration of the characteristics of the health system, political decisions, and cultural traits of the population (Barbagelata et al., 2020).

So, the COVID-19 pandemic has generated dissimilar psychological, social, and health difficulties in the different population groups analyzed.

This Research Topic aimed to collect evidence, through the results of different research teams in different countries, and according to the characteristics of the preventive measures taken by the health authorities of each place, about the effects of isolation and the fear of contagion by COVID-19, in different population groups: children, adolescents, the elderly, parents, healthcare workers, between others.

In this Research Topic we have collected thirty one Original Research articles (meta-analysis, transversal and cross cultural studies) that presents a comprehensive review about the psychological consequences of COVID-19 in topics including: Fear of COVID, Stress and lockdown, Effect on wellbeing; Post-traumatic stress, Effects on mental health, Vaccination, Parental influence and children behavior, Effects on anxiety, depression and loneliness, Prosociality, Cyberchondria, Burnout and stress, and Personality, in the populations groups: general population, college students, patients with schizophrenia, youths, adolescents, middle school students, university students, employees, teachers, healthcare workers, and sports players, from Israel, Spain, Argentina, Ecuador, México, China, Peru, Romania, Iran, Colombia, Italy, United Kingdom, and Pakistan, among others.

## Fear of COVID

Bitton and Laufer analyze whether greater resilience arises when someone is in permanent insecurity compared to exposure to an unknown threat such as COVID-19. Results indicated that Israelis living in a permanent conflict zone had similar levels of resilience as those not exposed to conflict. Luo et al. carried out a meta-analysis. They have reviewed a total of 44 articles with a sample size of 52,462 and concluded that in all the countries studied, the average fear of COVID was high. Therefore, it is important to consider the effects of COVID-19 on mental health. Rania and Coppola analyzed the answers of 500 Italian people, after the spread of the vaccination. The results showed that fear of COVID-19 produces emotional disturbance in the entire population, with young people feeling the most alone and the least admitting social distancing. Older people and those with high incomes are the ones with the least stress.

## Stress and lockdown

Rodriguez et al. found that women perceived more stress than men and the same occurs with people with low income or economic instability. On the other hand, lower stress has been found in couples without children isolated in residential zones. Older people and those with high incomes were the ones with the least stress. Li L. et al. carried out a study with a sample of 3,398 residents in China. The results indicated that there were several variables that influenced the application of strategies on home quarantine, such as gender, region, employment, depression, perceived social support, among others.

Huang et al. analyzed the effect of the COVID-19 on the feelings of 7–9 Chinese students during the confinement for the pandemic. They concluded that the students suffered feelings of loss of control and negative emotions that differed significantly according to demographic variables at different times during the pandemic.

## Effect on wellbeing

Dai et al. found that health and wellbeing were affected due to the COVID and lockdown measures and that Emotion Regulation intervention reduces the negative psychological impacts for improving quality of life. Tan et al. analyzed a sample of 1,871 Chinese students during the COVID-19 pandemic. The results indicated that resilience positively impacted on psychological wellbeing and that enhancing resilience mitigates the impact of environmental stress on psychological wellbeing. Tan et al. analyzed a sample of 1,871 Chinese students during the COVID-19 pandemic. The results indicated that resilience positively impacted on psychological wellbeing and that enhancing resilience mitigates the impact of environmental stress on psychological wellbeing. Boluarte-Carbajal et al. carried out a study with a Peruvian sample to evaluate socio-demographic variables and mental health during COVID-19 pandemic. They found that the fear of COVID-19, the Negative Affect and the Positive Affect influenced on the appearance of anxiety and depression. Yasmin et al. studied online a sample of 420 participants from Pakistan during COVID pandemic. The individuals informed in general that their mental health experienced a negative impact, have suffered family abuse, and have scored high on General Anxiety Disorder and low on wellbeing.

## Post-traumatic stress

Qiu et al. carried out a systematic review and meta-analysis and revised a total of 106 studies. They concluded that post-traumatic stress appears frequently among persons that suffer infectious disease outbreak and that it would be important to take preventive measures against post-traumatic stress.

## Effect on mental health

Li Y. et al. carried out a systematic review and meta-analysis based on about 27 articles with a total of 706,415 participants. They found that depression and anxiety strongly raised among college students during the COVID-19 pandemic. Zurlo et al. carried out a repeated cross-sectional survey with the objective of study the impact of the COVID-19 pandemic in Italian university students' customary life. The results indicated that psychopathological symptoms such as depression and phobic- anxiety, among others, have grown significantly since the beginning of the pandemic. At the same time, with the advance of the pandemic, psychological symptoms and stress due to COVID increased significantly. Silva Soares et al. carried out a cross-sectional study of 401 Brazilian physical sports and e-sports players, evaluating social connectedness, depression, anxiety, stress, and demographic variables. The main findings were: (1) social connection and mental health were significantly related in all the samples, (2) when the differences between the physical and e-sport samples were studied, only a difference in social connection and depression was found, (3) in the total sample a relationship was found between social connection with depression, anxiety, and stress.

## Vaccination

Iacob et al. studied the vaccination intention in a sample of 864 adults from Romania and concluded that vaccination intention was directly predicted by fear of the pandemic and indirectly by the perceived threat of getting sick and the benefits of vaccination. Caycho-Rodríguez et al. explored conspiracy beliefs about COVID-19 and the respective vaccine in thirteen Latin American countries. They found greater conspiracy ideas in women, less educated people, and those who were informed about the vaccine from family and friends.

## Parental influence and children behavior

Khozaei and Carbon  analyzed the effect of parental stress on children's physical activity and wellbeing in a sample of Iranian children and their parents. They concluded that parents with greater stress and more restrictive put their children at risk of having alterations in their mental health. Kurata et al. conducted an online survey in three Asian countries and in the United States to assess parental stress, anxiety, and fear related to COVID-19. The results indicated that parental stress had significantly increased during the pandemic in all the countries studied. Vargas Rubilar et al. studied 646 mothers of school-age children in Argentina during the COVID-19 lockdown. They found that the mothers presented moderate stress and that it was not caused only by the pandemic but by other contextual variables, such as the number of children, among others.

## Effect on anxiety, depression, and loneliness

Shen et al. conducted a study in a sample of 2,361 residents and indicated that anxiety and depression in the period of low transmission were potential factors for long-term depression and anxiety for some residents. Zhang et al. analyzed how coping style mediates in the relation between loneliness and depression and how gender mediates in the relation between loneliness and coping styles during the COVID-19 pandemic. They studied 337 Chinese college students during the COVID-19 lockdown and found that less loneliness means less depression, and that positive coping prevents depression and loneliness. Mei et al. studied 1,414 Chinese company employees, through a 1-year longitudinal study during the COVID pandemic. The results indicated the existence of a dynamic relationship between social support and mental health over time, that social support predicted the appearance of depressive symptoms and interpersonal sensitivity, and that depression predicted social support. Chocho-Orellana et al. carried out a cross-sectional study through an online survey in an Ecuadorian and Spanish sample. The results indicated significantly more depression, anxiety and stress after quarantine in Ecuadorians. Second, public prosociality, lower stress as challenge, higher stress as threat and empathy are predictors of depression and anxiety. Finally, in both countries depression, anxiety and stress increased after the lockdown. Burkova et al. studied a sample of 15,375 participants from 23 countries during the COVID pandemic. They found that gender, country, and personal aspects significantly influenced anxiety, and that people from countries with higher anxiety perceived the pandemic as more dangerous. Those who trusted state authorities presented lower levels of anxiety.

## Prosociality

Mesurado et al. analyzed the efficacy of a short, online intervention program (Hero Program) during the lockdown due to COVID-19, to increase the positive emotions and prosocial behavior of Colombian adolescents. They indicated that Hero Program was efficacious for promoting joy, gratitude, serenity, and personal satisfaction and that these emotions predisposed Colombian adolescents to act prosocially.

## Cyberchondria

Peng et al. investigated the status and influencing factors of cyberchondria (the anxiety-amplifying effects of online health-related searches) in 674 residents in China during the COVID-19 epidemic. Their findings showed that nearly a quarter of the participants scored high in cyberchondria during the pandemic, being health anxiety and COVID-19-related online information-seeking behavior, including online duration, topics and choice on different information channels, important influencing factors of cyberchondria.

## Schizophrenia

Caqueo-Urizar et al. have analyzed the psychosocial effects of the COVID-19 pandemic on 120 Chilean patients with schizophrenia, and their caregivers. The results showed that patients with schizophrenia who had been in quarantine for almost a year showed similar levels of concern as their caregivers in the domains of health and social life. However, caregivers showed significant differences from patients in the areas of income, concern, and employment status. In addition, patients who were infected with COVID-19 showed lower levels of wellbeing and worse psychological recovery.

## Healthcare workers

Richaud et al. analyzed the difference in psychological distress of the healthcare workers in three different periods of the COVID-19 pandemic in Argentina, through a repeated cross-sectional online survey. The results indicated differences between the evaluated periods. Perceived concerns about the possibility of infecting loved ones and infecting themselves were greatest in the periods after the onset of the pandemic. In addition, the perception of how the work environment worsened and how lack of sleep interfered with their work, the same as depression, anxiety, and intolerance of uncertainty were also higher in periods 2 and 3. Finally, the indicators of high tension and concurrent lack of emotional control, which was greater in the last periods evaluated, were also expressed in the coping strategies.

## Burnout and stress

Vargas Rubilar and Oros carried out a study with 9,058 Argentine teachers, who had to complete online self-report measures, during the COVID-19 pandemic. The results indicated that more than 60% of the educators reported high and moderately high levels of stress. The more stress they perceived, the higher the manifestation of unwanted psychophysical symptoms. Professional burnout was higher for teachers with a higher load of stress and with more psychophysical indicators of discomfort. Pelly et al. performed a longitudinal study to examine the wellbeing of 621 full-time workers assessed before and during the first lockdown in the United Kingdom. Overall, levels of stress, self-rated mental health, positive emotions and life and job satisfaction are not adversely affected by the restrictions. There is a reduction in the burnout symptoms of disengagement and exhaustion and in the frequency with which negative emotions are experienced at work. Workers gained autonomy, they were closer to their co-workers, and more engaged to their organizations, but their home-life was more unsatisfactory.

## Personality

Cirimele et al. studied using a person-oriented approach, the relation of personality profiles (positivity, irritability, and hostile rumination) and the ability to express positive emotions and regulating anger emotion, with adaptive and maladaptive outcomes during the first Italian lockdown. They administered an online survey and included 1,341 participants living in Italy. Overall, the findings evidenced the existence of three different profiles (i.e., Resilient, Vulnerable, and Moderate) and, especially for the vulnerable profile and young adults, a greater maladaptive consequence of the quarantine.

## Author contributions

This Research Topic Psychosocial effects of isolation and fear of contagion of COVID-19 on the mental health of different population groups was proposed by MR. As editors, all authors worked collaboratively to decide which manuscripts were accepted, and each of them was subject to review by the team of editors as well as peer reviewers. All authors contributed to the article and approved the submitted version.

## Conflict of interest

The authors declare that the research was conducted in the absence of any commercial or financial relationships that could be construed as a potential conflict of interest.

## Publisher's note

All claims expressed in this article are solely those of the authors and do not necessarily represent those of their affiliated organizations, or those of the publisher, the editors and the reviewers. Any product that may be evaluated in this article, or claim that may be made by its manufacturer, is not guaranteed or endorsed by the publisher.
